# Alkaline Activation of Binders: A Comparative Study

**DOI:** 10.3390/ma17030667

**Published:** 2024-01-30

**Authors:** Bianca Ignacio Almeida Alves, Markssuel Teixeira Marvila, José Alexandre Tostes Linhares Júnior, Carlos Maurício Fontes Vieira, Jonas Alexandre, Afonso Rangel Garcez de Azevedo

**Affiliations:** 1 LAMAV—Advanced Materials Laboratory, UENF—University of the Northern Rio de Janeiro, Av. Alberto Lamego, 2000, Campos dos Goytacazes 28013-602, Brazil; bianca.ig.almeida@gmail.com (B.I.A.A.); tosteslinhares@gmail.com (J.A.T.L.J.); vieira@uenf.br (C.M.F.V.); 2 Rio Paranaíba Campus, UFV—Federal University of Viçosa, Rodovia BR 230 Km 7, Rio Paranaíba 38810-000, Brazil; markssuel.marvila@ufv.br; 3 LECIV—Civil Engineering Laboratory, UENF—State University of the Northern Rio de Janeiro, Av. Alberto Lamego, 2000, Campos dos Goytacazes 28013-602, Brazil; afonso@uenf.br

**Keywords:** metakaolin, blast furnace slag, fly ash, geopolymer, alkaline activation, Portland cement

## Abstract

Binders formulated with activated alkali materials to replace Portland cement, which has high polluting potential due to CO_2_ emissions in its manufacture, have increasingly been developed. The objective of this study is to evaluate the main properties of activated alkali materials (AAM) produced by blast furnace slag, fly ash, and metakaolin. Initially, binders were characterized by their chemical, mineralogical and granulometric composition. Later, specimens were produced, with molarity variation between 4.00 and 5.50, using the binders involved in the research. In preparing the activating solution, sodium hydroxide and silicate were used. The evaluated properties of AAM were consistency, viscosity, water absorption, density, compressive strength (7 days of cure), calorimetry, mineralogical analysis by X-ray diffraction, and morphological analysis by scanning electron microscopy. The results of evaluation in the fresh state demonstrate that metakaolin has the lowest workability indices of the studied AAM. The results observed in the hardened state indicate that the metakaolin activation process is optimized with normal cure and molarity of 4.0 and 4.5 mol/L, obtaining compressive strength results after 7 days of curing of approximately 30 MPa. The fly ash activation process is the least intense among the evaluated binders. This can be seen from the absence of phases formed in the XRD in the compositions containing fly ash as binder. Unlike blast furnace slag and metakaolin, the formation of sodalite, faujasite or tobermorite is not observed. Finally, the blast furnace slag displays more intense reactivity during thermal curing, obtaining compressive strength results after 7 days of curing of around 25 MPa. This is because the material’s reaction kinetics are low but can be increased in an alkaline environment, and by the effect of temperature. From these results, it is concluded that each precursor has its own activation mechanism, observed by the techniques used in this research. From the results obtained in this study, it is expected that the alkaline activation process of the types of binders evaluated herein will become a viable alternative for replacing Portland cement, thus contributing to cement technology and other cementitious materials.

## 1. Introduction

Provis [1] points out that alkali-activated materials present a real possibility of enhanced sustainability in civil construction. This is because these materials can replace Portland cement, a material used on a large scale in civil construction which can emit an enormous amount of CO_2_ in its production. This information has already been widely discussed in other studies published internationally [2].

With the decrease in the use of natural resources as raw material, eco-friendly proposals are being introduced in the ceramic coating sector or as substitutes for cementitious materials based on Portland cement, expanding the possibilities for the use of waste and by-products. This can reduce costs in the production process and ensure that the disposal of these wastes is done correctly. On the other hand, the use of alkali-activated materials to replace Portland cement allows the reuse of wastes otherwise unused by industries, due to their properties. One definition of these materials is an inorganic polymer that is synthesized through alkaline activation, based on aluminosilicates or calcium materials [3,4].

The synthesis of activated alkali materials is carried out by activating aluminosilicates at different temperatures, where metakaolin is often used as a precursor and hydroxides and sodium or potassium silicates as activators [5]. They are resistant to high temperatures and acid degradation, also having good mechanical properties, which depend on the alkaline cation Na^+^, the SiO_2_/Al_2_O_3_ molar ratio, and the circumstances in which the reaction takes place [6]. Therefore, they offer a great alternative to Portland cement. The use of metakaolin in alkali-activated materials may raise doubts regarding the ecological potential of the material, since obtaining this binder requires a calcination step with heat release, in contrast to other materials such as blast furnace slag and fly ash. However, several studies, such as Kul et.al [7] and Munir et al. [8], performed a life cycle analysis comparing activated alkali concrete produced with metakaolin and Portland cement-based materials. In conclusion, the authors highlight that materials based on alkali-activated metakaolin had 60% lower CO_2_ emissions than Portland cement materials. This proves the ecological potential of using metakaolin as a substitute for Portland cement.

Other precursors commonly considered in international surveys are fly ash and blast furnace slag, although they are commonly used together with other precursors [9,10]. This raises the question: how does the individual activation process of these materials occur? This is the question that we will answer in this article.

One advantage of using activated alkali materials in concrete production is that the CO_2_ emissions are about 3.6 times lower than in conventional concrete production. The synthesis of these materials becomes more sustainable when cleaner raw materials are used to produce sodium silicate: alkaline activators based on potassium silicate emit about 1.1 ton/m^3^ of CO_2_, whereas activators based on alternative sources emit approximately 572.3 kg/m^3^ [11,12].

One point that needs to be highlighted is the common pattern adopted in recent years of carrying out research with more than one precursor. An example is Gorhan and Kurklu [13], who addressed in their research the use of fly ash from a thermoelectric industry in Turkey in partial replacement of metakaolin. The mortar was made and placed in prismatic molds with varying molarity (9 M, 12 M and 15 M), thus using three different ratios of metakaolin substitution with three different molarities. The results obtained by the authors highlight that the NaOH factor does not influence so much the physical properties like density and water absorption of the mortars produced, but has a significant impact on the compressive strength of the material. Elevated temperatures and long curing time became unnecessary and the best compression results were obtained at 9 M molarity, as at 15 M molarity the compressive strength was small. Therefore, the best partial replacement range is 60%.

Other examples of work that evaluated the combined effect of binders on alkaline activation include Peng et al. [14] who evaluated the preparation of activated alkali materials (geopolymers) based on metakaolin and fly ash for passive fire protection, proving that this type of material is fire resistant. Tian et al. [15] evaluated the production of activated alkali materials (geopolymers) produced with metakaolin and fly ash from municipal solid waste incineration. Carvalho et al. [16] evaluated the production of activated alkali materials (geopolymers) in mortars using metakaolin and biomass fly ash as binders. Based on this, we can see that the combined mechanism of these binders is known. But how does this mechanism happen in isolation, considering only metakaolin or only fly ash?

Therefore, the main objective of this article is to evaluate the alkaline activation process of classic binders, such as metakaolin, blast furnace slag, and fly ash. The main novelty of the research is the individual evaluation of the binders and their activation mechanisms, since, in general, the alkaline activation process is carried out together for blast furnace slag and fly ash. Therefore, the individual investigation of alkaline activation of the different binders evaluated in this research (metakaolin, blast furnace slag, and fly ash) constitutes an important innovation that justifies the publication of the article.

## 2. Materials and Methods

The materials that were used in the study were metakaolin, blast furnace slag, fly ash, and a water/sodium hydroxide/silicate solution from Nox Lab Solution (Mauá, Brazil). The metakaolin used in the research is one of the most abundant raw materials and can be used in the production of activated alkali materials [17]. This material is obtained from kaolin through heat treatments, forming an amorphous structure making it more reactive, and its use is desirable due to its high dissolution rate when found in an alkaline medium and the control it exerts in the reaction [18]. Blast furnace slag is a co-product generated in the production of pig iron, presenting amorphism and reactivity when the cooling process is rapid and abrupt. It is used as a supplementary cementitious material and is highly viable for alkaline activation [19]. Fly ash is a product originating in plants that burn coal to obtain energy, characterized by high fineness and lightness [20].

Each binder used in the research was produced from an initial quantity of 5 kg of material in powder form. The materials were dried in an oven and sieved through a 100-mesh sieve to standardize particle size. The materials were obtained from the industrial sectors responsible for generating each binder, such as the steel industry (blast furnace slag), thermoelectric industry (fly ash), and HP Ultra (metakaolin) (Jundiaí, Brazil). The approximate costs of each binder used in the research are (prices based on the Brazilian market) [21]: metakaolin costs approximately 0.52 USD/kg; blast furnace slag costs 0.40 USD/kg; and fly ash costs 0.35 USD/kg. By way of comparison, the costs of Portland cement are approximately 0.80 USD/kg, demonstrating the feasibility and availability of using the binders described in this research.

The binders used were characterized by X-ray fluorescence (XRF), using Axios Max equipment from Malvern Panalytical (Malvern, UK). They were also characterized through X-ray diffraction (XRD) using a 6000 diffractometer from Shimadzu (Kyoto, Japan), using a copper source (Cu-Kα), voltage of 40 kV and 2θ sweep ranging from 10° to 60°. Subsequently, the binders were evaluated for laser granulometry using Malvern Mastersizer 2000 equipment (Malvern, UK), applying 15 s of ultrasound with an ultrasonic displacement of 12.5 according to the equipment scale. As activating solution, microbeads of sodium hydroxide (99% purity) and sodium silicate, with a silica modulus of 1.5, were used. The evaluated compositions were dosed according to the molarity of the activator solution, ranging from 4.0–5.5 mol/L, as shown in Table 1. It should be noted that the quantities described in Table 1 are evaluated based on molarity and not based on mass ratio. The research was carried out using paste, therefore sand was not used as fine aggregate. Furthermore, as the objective of this research is to evaluate the alkaline activation of different precursors, Portland cement was not used in the research.

The activating solution was produced 24 h in advance using a magnetic mixer. Mixing of the activated alkali material was performed using a mechanical mixing equipment from Solotest (São Paulo, Brazil) with a total mixing time of 90 s. Consistency and viscosity properties were evaluated [22] whilst the material was in its fresh state. Cylindrical specimens of 50 × 100 mm were molded, using 3 samples for each test and each composition. The specimens were molded using 3 compaction layers, with 25 blows per layer following normative procedures [23]. After molding, the material was cured in one of two ways: (i) at a normal temperature of 25 °C; or (ii) thermally at a temperature of 65 °C for a period of 7 days, with temperatures maintained throughout the curing time. Mass density and water absorption tests [24] were carried out, in addition to compressive strength tests [23]. In this test, an INSTRON model 5582 universal testing machine (Norwood, MA, USA) was used, with a loading rate of 0.5 mm/min.

After analyzing the fresh and hardened state results, especially the compressive strength results, a complementary analysis was carried out on the composition with the best results. The composition with molarity of 4.5 mol/L was chosen. A calorimetry test was carried out, using the procedure of the American standard [25] using a calorimeter composed of a constant speed stirrer, insulating wooden box, Dewar flask, Beckman differential thermometer (Jiangsu, China), and funnel. A total test time of 48 h was used, in line with other works consulted [26,27]. An X-ray diffraction test (XRD) was carried out under the same conditions previously highlighted in the analysis of binders, and a scanning electron microscopy (SEM) test was carried out using a Jeol microscope model JSM 6460 LV (Peabody, MA, USA). The samples used in the XRD test were sieved through a 200-mesh sieve. The samples tested in SEM were cut with the aid of a saw and isopropyl alcohol as a lubricant. They were sequentially dried in ovens and fixed to the sample holder with a piece of double-sided silver tape and metallized with gold.

## 3. Results and Discussion

Table 2 presents the chemical composition of the binders used in the research. An important parameter is the CaO/(SiO_2_ + Al_2_O_3_) ratio, as highlighted by Marvila et al. [28]. This ratio is 0.07 for fly ash, 0.001 for metakaolin, and 1.11 for blast furnace slag. This differentiates these binders from each other. Fly ash and metakaolin are considered binders rich in aluminosilicates, potentially considered pozzolanic materials. The alkaline activation process of these products originates geopolymers, or inorganic polymers [29,30]. Blast furnace slag, on the other hand, is considered a precursor rich in calcium, due to the ratio being greater than 1. In general, these materials are cementitious, but with much lower reactivity than Portland cement. The slag activation process takes place in alkaline media, where binder hydration is accelerated [31]. In short, calcium-rich binders react in a similar way to hydration, but with increased kinetics.

Another highlight in Table 2 is the Fe_2_O_3_ content. It is known that this component, in amounts greater than 5%, may represent the formation of ferrosialates [32,33], a type of geopolymer different from that formed by the reaction of SiO_2_ and Al_2_O_3_. These products are expected to form in the fly ash reaction, as the observed content is 6.5%. The amount of loss on ignition for the binders used in the research is not indicated in Table 2 because the quantities are very low, such as: 1.88% for metakaolin, 0.82% for fly ash, and <0.3% for blast furnace slag.

**Figure 1:**
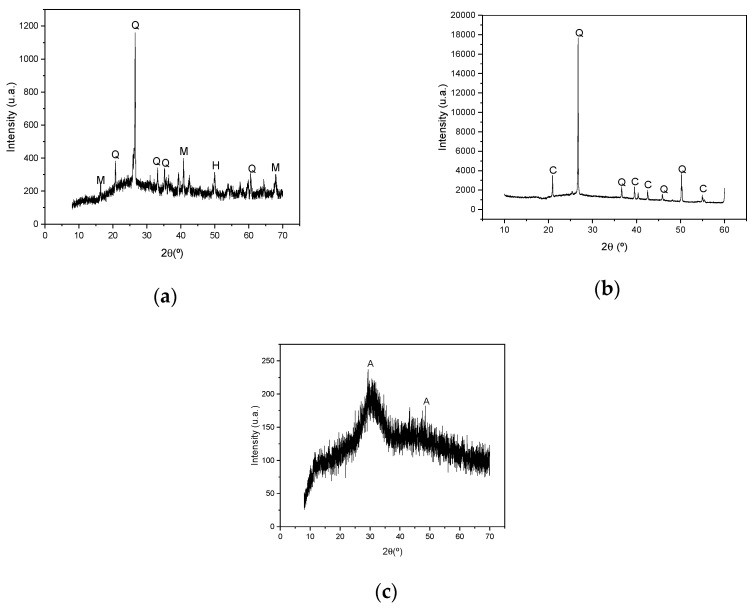
XRD of binders: (**a**) fly ash; (**b**) metakaolin; (**c**) blast furnace slag. Legend: Q = quartz; M = mullite; H = hematite; A = akermanite; C = kaolinite.

Figure 1 shows the results of the mineralogical analysis by XRD of the binders evaluated in the research. Note the presence of quartz particles in the mineralogy of metakaolin and fly ash. This can harm the activation process of the binders, since a high degree of amorphism is required for the material to be activated correctly [34,35]. Amorphous bands are observed in these two binders, especially in the range of 20° to 30° and 40° to 50° for metakaolin and in the range of 20° to 35° for fly ash. This information is promising for alkaline activation. Blast furnace slag, on the other hand, has a high amorphism content, indicating that it is a material compatible with the activation process. Thus, the prognosis indicates that although all binders have amorphism, the quartz peaks in metakaolin and fly ash can impair the performance of these materials. It is known that the silica present in binders can be amorphous or crystalline [36]. When silica crystallizes, it is likely to form quartz particles, reducing the amorphism of the binder.

**Figure 2:**
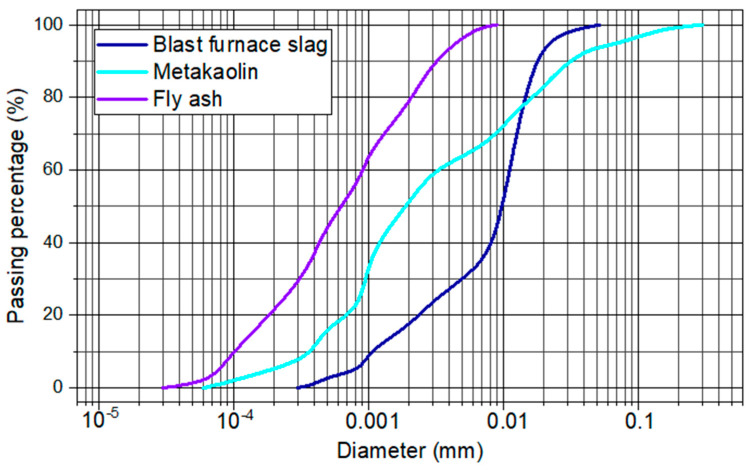
Granulometry of the binders.

Figure 2 shows the granulometry results of the evaluated binders. Fly ash has a finer grain size than metakaolin and blast furnace slag. This is indicated by the maximum characteristic dimension (MCD), which is approximately 0.003 mm for fly ash, while it is 0.02 mm for slag and 0.03 mm for metakaolin. Another important point to be highlighted is that metakaolin has a more continuous granulometry than blast furnace slag [2,37]. This indicates that metakaolin has a greater tendency to contribute to the compaction of the hardened material when compared to blast furnace slag. The same can be said for fly ash. Because it is thinner, it has a greater tendency to reduce porosity considering only the physical aspects.

**Figure 3:**
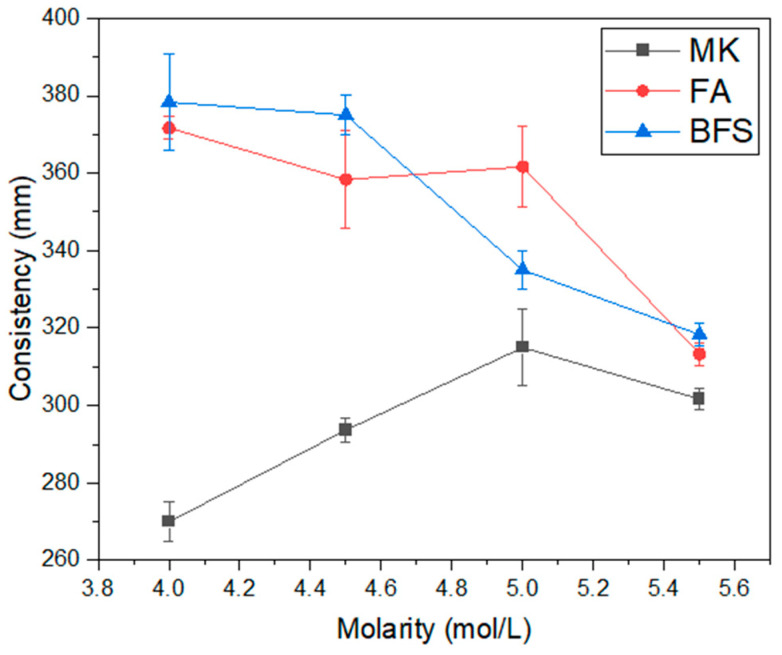
Consistency of geopolymers.

**Figure 4:**
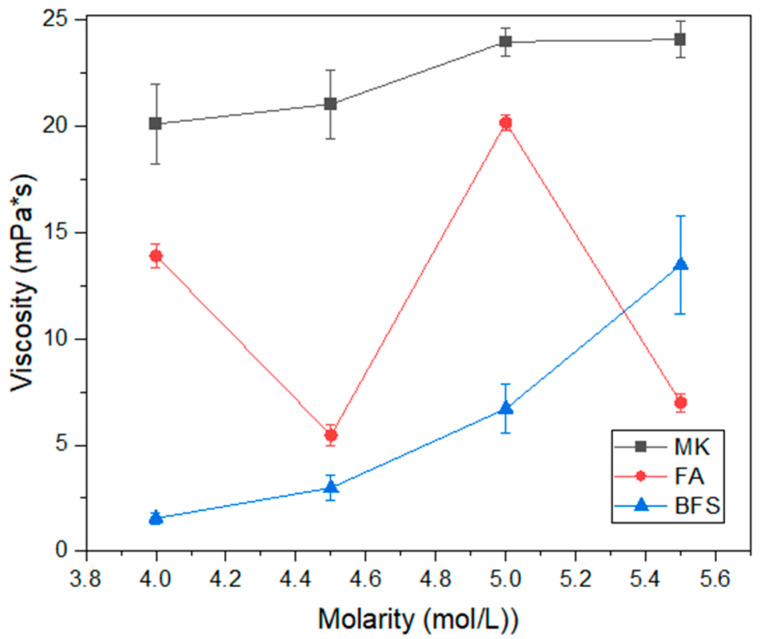
Viscosity of geopolymers.

Figure 3 shows the consistency results of the studied alkali-activated materials, while Figure 4 shows the viscosity results. It is observed that the material with the same consistency is metakaolin-based, which also has a higher viscosity, around 20–24 mPa·s. This indicates that metakaolin-based compositions have lower workability compared to those with fly ash or blast furnace slag as precursor. The higher viscosity of the composition containing metakaolin, previously reported by other authors [38,39], also indicates that the material has greater reaction kinetics, since it undergoes a gel formation process within a few minutes [40]. This is related to the high reactivity of metakaolin, as reported in other studies [6], and indicates a tendency for the material to present good resistance parameters at less advanced ages. Regarding the influence of molarity on viscosity, for compositions containing metakaolin, the standard trend is observed, that is, the higher the molarity of the solution, the higher the viscosity of the activated alkali material [41,42]. This can even impair the compaction of the material in the hardened state.

The compositions with blast furnace slag and fly ash have equivalent consistencies, considering the margin of error, for molarities of 4.00, 4.50 and 5.50 mol/L. There is also a tendency for these binders to exhibit reduced workability as the activator solution molarity increases. This is an expected result, already reported in previous studies [31,43]. The viscosity of compositions containing fly ash does not show a clear trend. This is related to the granulometry of the material. As fly ash is finer, the effect of molarity on the viscosity property is inconclusive. The compositions containing blast furnace slag showed the expected pattern for viscosity, that is, there is an increase in this property as the molarity of the activator solution increases. The results increase from 1.56 mPa·s at 4.00 mol/L molarity to 13.46 mPa·s at 5.50 mol/L molarity. This represents an 8.62× increase in material viscosity.

**Figure 5:**
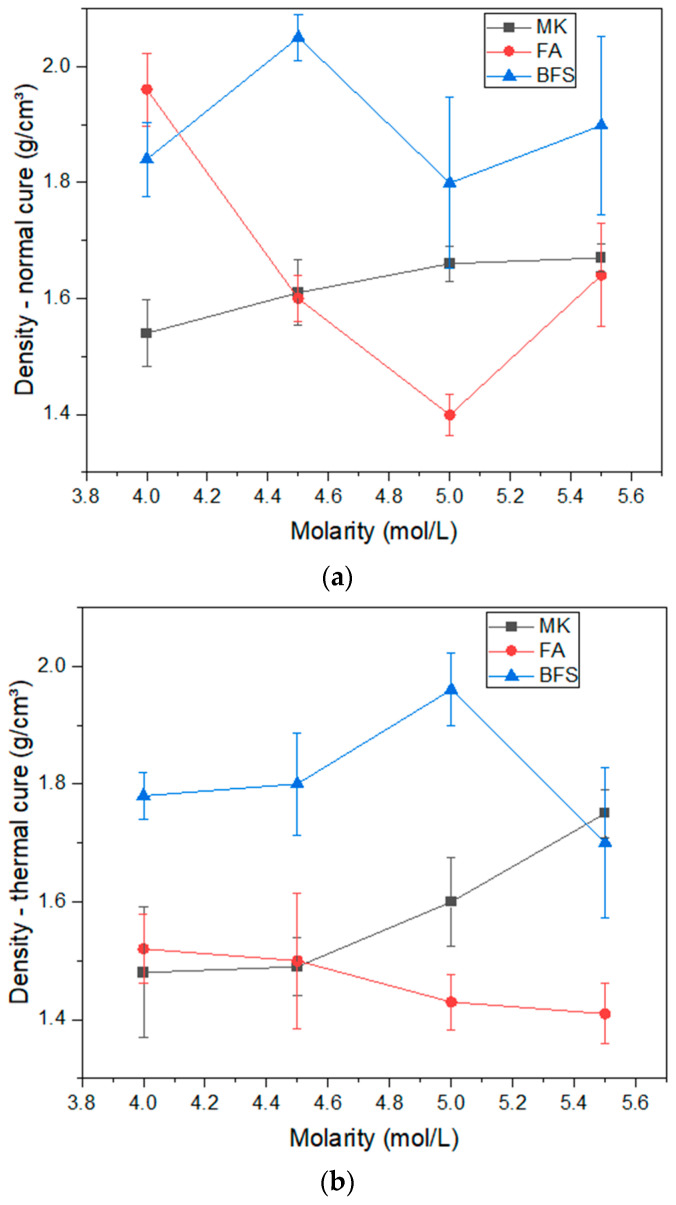
Density of geopolymers: (**a**) normal cure; (**b**) thermal cure.

Figure 5 shows the density results obtained in normal and thermal curing. In normal curing, the compositions with blast furnace slag presented densities varying between 1.8 and 2.0 g/cm^3^. The compositions containing metakaolin presented densities varying between 1.5 and 1.6 g/cm^3^. In these two compositions there was no clear effect of molarity on material density. The composition with fly ash showed a density reduction of 1.9 g/cm^3^ for the molarity of 4.00 mol/L to values around 1.4 g/cm^3^ in the compositions with molarity of 5.00 mol/L. This reduction in density indicates increased porosity, as will be discussed in relation to the water absorption and compressive strength results. Therefore, for normal curing, it is possible to observe that the compositions with metakaolin are less dense than the compositions with blast furnace slag, which presents densities comparable with Portland cement in other research, e.g., Sinkhonde and Mashava [44] who obtained densities between 1.9 and 2.05 g/cm^3^ for different cementitious pastes.

Density results in thermal curing are lower than normal curing. It is observed that the compositions with metakaolin were in the range of 1.4 to 1.6 g/cm^3^, while the compositions with blast furnace slag reduced to the range of 1.75 to 1.90 g/cm^3^. The reduction in density through thermal curing has already been reported in other studies and is due to the elimination of water at younger ages, enhanced by the thermal effect [45]. Therefore, the results obtained are comparable with other studies.

**Figure 6:**
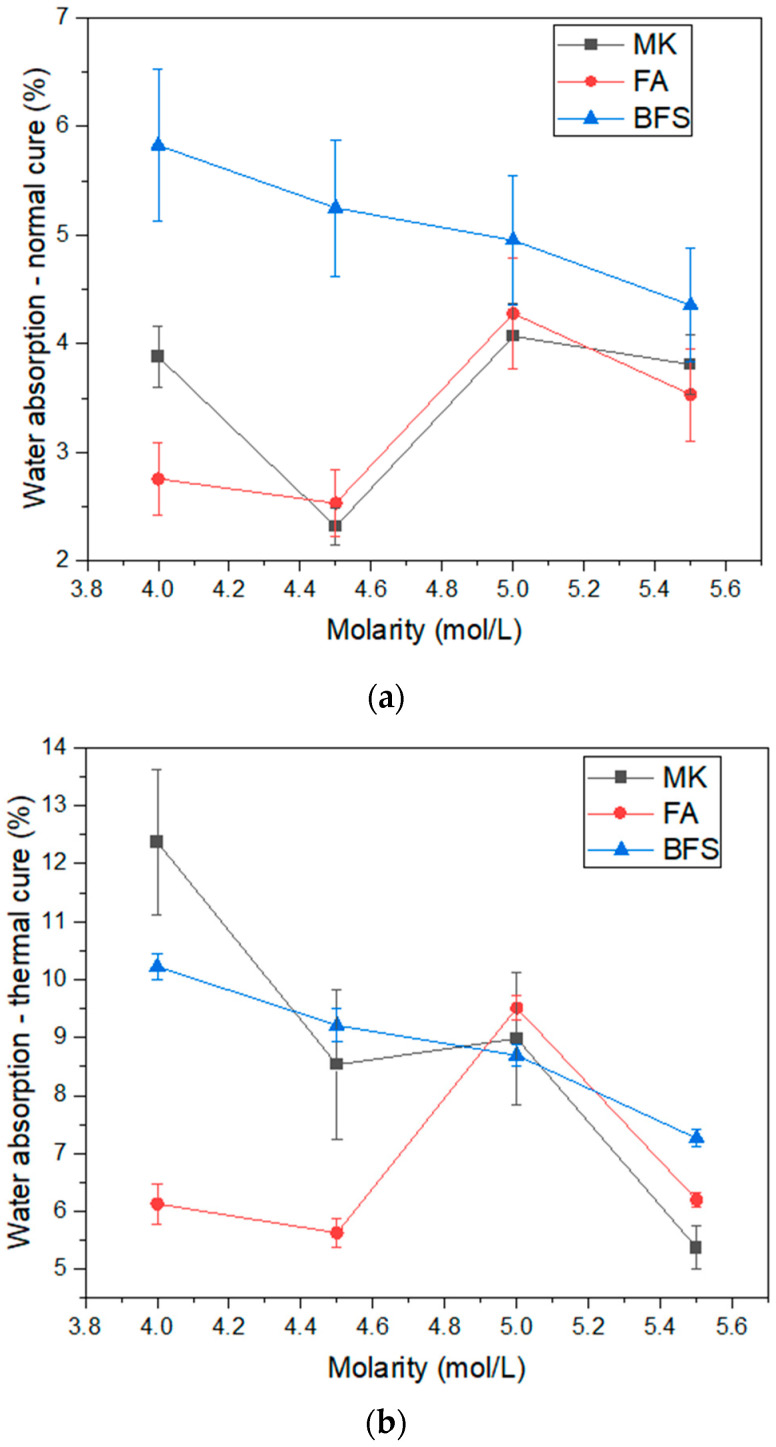
Water Absorption of geopolymers: (**a**) normal cure; (**b**) thermal cure.

Figure 6 shows the water absorption results obtained in normal and thermal curing. In normal curing, it is observed that blast furnace slag has the highest water absorption values, ranging from 5.83% to 4.95%. This indicates that this is the composition with the highest porosity among the evaluated binders, which does not necessarily imply the composition with the lowest resistance, since the resistance mechanisms of activated alkali materials are not only physical [46]. For compositions with blast furnace slag, water absorption tends to reduce as material molarity increases. In the compositions containing fly ash and metakaolin, there is no clear trend in the relationship of water absorption and molarity. Note that, except for the composition with molarity of 4.00 mol/L, there is equivalence in water absorption in normal curing for the compositions with fly ash and metakaolin. This may be related to the granulometry of the material, since fly ash is finer than other binders, while metakaolin has continuous granulometry, different from blast furnace slag, for example. That is, in normal curing, the water absorption results are justifiable by the granulometry results, being directly related to compactness and porosity in the hardened state. This is a pattern reported by other authors [47].

In thermal curing, there was an increase in water absorption of all evaluated compositions. This is related to the excessive water loss promoted by this type of cure. The same trend of reduction of water absorption with increasing molarity in the compositions with blast furnace slag is observed. The trend observed in normal curing is the same as for thermal curing in compositions containing fly ash. That is, the composition with a molarity of 4.5 mol/L presents less water absorption than the others, both in normal and thermal curing. As discussed in the density results (Figure 5), this is directly related to the porosity reduction for this composition and is a direct effect of the particle size of the fly ash, which is finer than the other binders. If the principles of resistance gain were only physical, it would be expected that compositions with fly ash would be more resistant. However, in alkali-activated materials, the resistance principle is also chemical [46,48], as will be discussed in relation to the compressive strength results. It is worth noting that the behavior observed for metakaolin is very different in normal and thermal curing. In normal curing, the water absorption values were all lower than the composition with blast furnace slag. For example, in the composition with molarity 4.00 mol/L, MK—4.0 showed an absorption of 3.88%, while BFS—4.0 was 5.25%. In thermal curing, this behavior is reversed: the MK—4.0 composition presents an absorption of 12.37%, while the BFS—4.0 composition became 10.22%. This drastic increase in water absorption, above expectations, even surpassing the composition with blast furnace slag, is directly related to the high reactivity of metakaolin. This precursor has a lamellar morphological pattern, which is responsible for its high specific surface area and consequently high contact region between binder and activator solution [18,49]. As alkaline activation reactions are essentially contact reactions, the fact that metakaolin has greater contact favors its reactivity. Thus, it is noteworthy that for metakaolin, thermal curing is not necessarily advantageous, since it favors the occurrence of porosity, a critical defect for ceramic materials.

**Figure 7:**
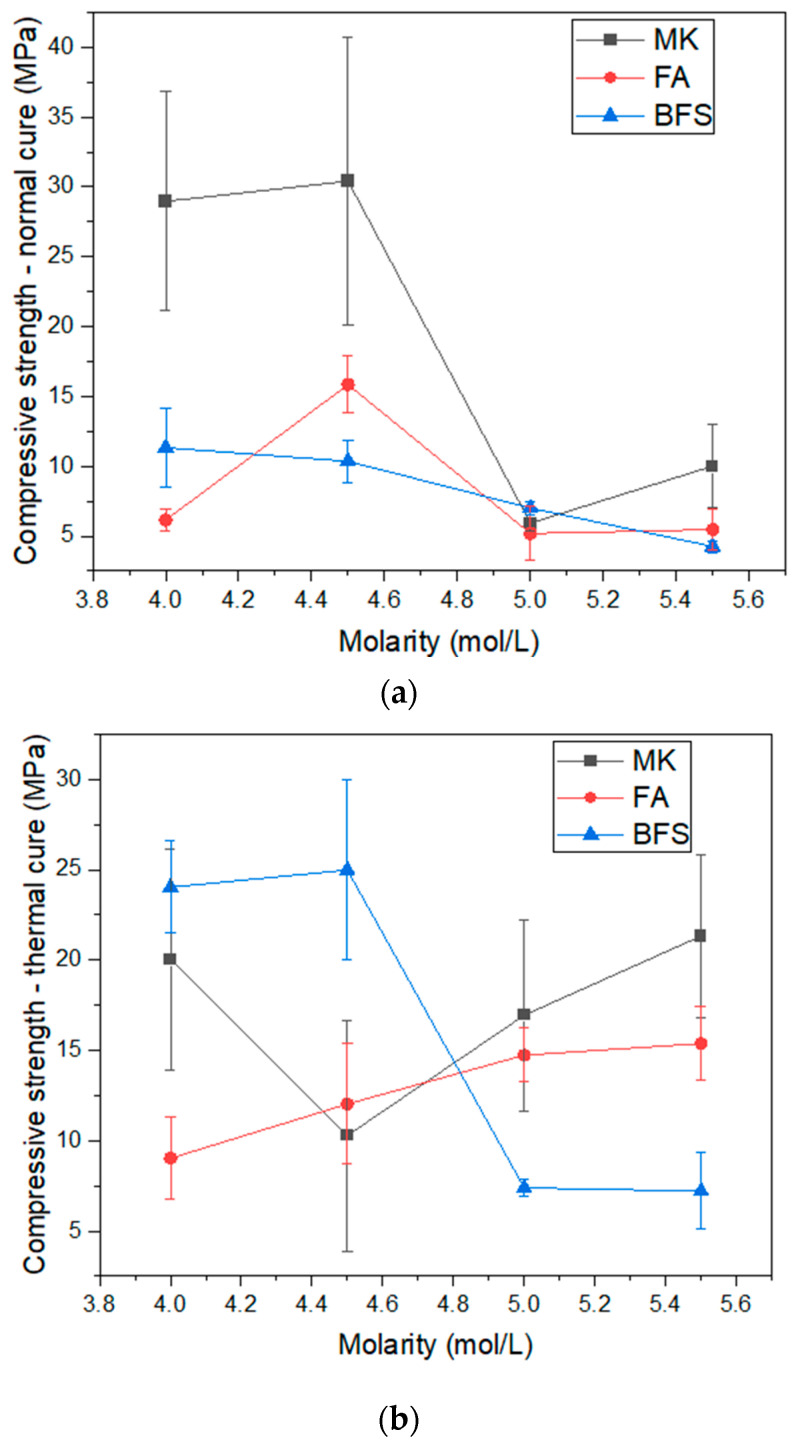
Compressive Strength of geopolymers: (**a**) normal cure; (**b**) thermal cure.

Figure 7 presents the compressive strength results obtained in normal and thermal curing. In normal curing, the best compressive strength values at 7 days were for the compositions with metakaolin at 4.00 and 4.50 mol/L, rated at 28.96 and 30.40 MPa, respectively. The high reactivity of the material does not favor resistance gain in thermal cure [18,49], where the values drop to 20.02 and 10.29 MPa, in the same compositions. These values are consistent with the results observed in water absorption and density. In normal curing, metakaolin compositions containing 5.00 and 5.50 mol/L show a drop in resistance, related to the high viscosity of the material. In thermal curing, for these same compositions, the activation kinetics is increased, representing a gain in resistance, but still not surpassing the compressive strength values in normal curing for the 4.50 mol/L composition. Therefore, it is understood that in the alkaline activation of metakaolin, due to the high reactivity of the material, thermal curing is not justifiable as it induces porosity defects reducing its resistance. It is also not justifiable to use activator solutions with molarities of 5.00 and 5.50 mol/L due to the high viscosity of the solutions, which impairs the behavior of the material.

The compressive strength results for blast furnace slag and fly ash in normal cure are between 5 and 15 MPa and show a similar trend. Overall, this indicates low alkaline activation activity. In thermal curing, however, the results of the two compositions are clearly different: while the blast furnace slag benefited from the thermal effect, with compressive strength increasing from 10.35 to 24.98 MPa in the 4.50 mol/L composition, the same did not happen in compositions with fly ash. Blast furnace slag is a precursor with cementitious characteristics. This means that the material has the power to agglomerate in the presence of water, but this happens at very slow rates. When used together with Portland cement, the alkaline medium favors the slag reaction process [50]. This is the same principle used in activation, where alkaline products, sodium hydroxide and silicate are used to accelerate the reaction of the slag, forcing its dissolution. The thermal effect increases the degree of molecular agitation of the dissolved products, contributing to an increase in the contact area and consequently resulting in resistance gain.

The compositions containing fly ash did not present an effective gain in resistance, when compared to the other binders studied in the research. It is worth mentioning that, although the compositions with fly ash have lower water absorption values than the compositions with blast furnace slag, this does not imply greater resistance. In other words, even though fly ash promotes greater compactness, it does not necessarily produce greater mechanical strength, even if it has smaller grain sizes. This indicates that the fly ash principle is more physical than chemical, which is why it is used in conjunction with other binders in several studies [51,52]. Although it has some reactivity, fly ash helps to reduce porosity more than chemical mechanisms, as will be highlighted through subsequent results.

To validate the discussion of the results presented in Figure 7, a statistical analysis was carried out using ANOVA (*p* ≤ 0.05). The analysis was carried out separately for each type of cure performed (normal and thermal) to simplify the results obtained. The analysis was carried out using a randomized block design, where the blocks represent the different types of binders used in the research (MK, FA, BFS) and the treatments represent the different molarities studied (4.0 to 5.5 mol/L). Regarding Normal Cure (Table 3), there are statistical differences in blocks and treatments (F test > F standard). This indicates that the evaluated binders (blocks) present a significant difference in compressive strength, as well as significant differences in treatments, that is, molarity directly interferes with the compressive strength results too. Regarding Thermal cure (Table 4), there are statistical differences in the blocks and treatments (F test > F standard), similar to Normal cure. This indicates that there are statistical differences in the evaluated binders (blocks) and molarity (treatment) for the compressive strength parameter in Thermal Cure. Based on this, it is possible to conclude that the discussions presented in the previous sections are valid.

**Figure 8:**
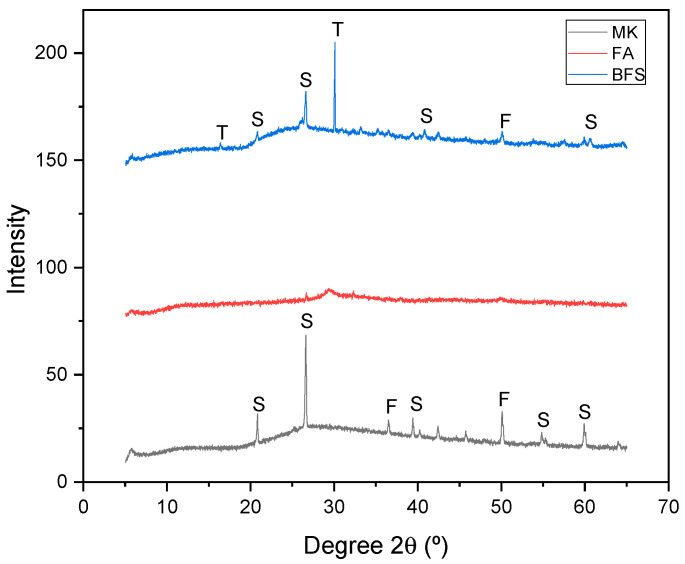
X-ray diffraction (XRD) of composition of 4.5 mol/L. Legend: S = sodalite; F = faujasite; T = tobermorite.

**Figure 9:**
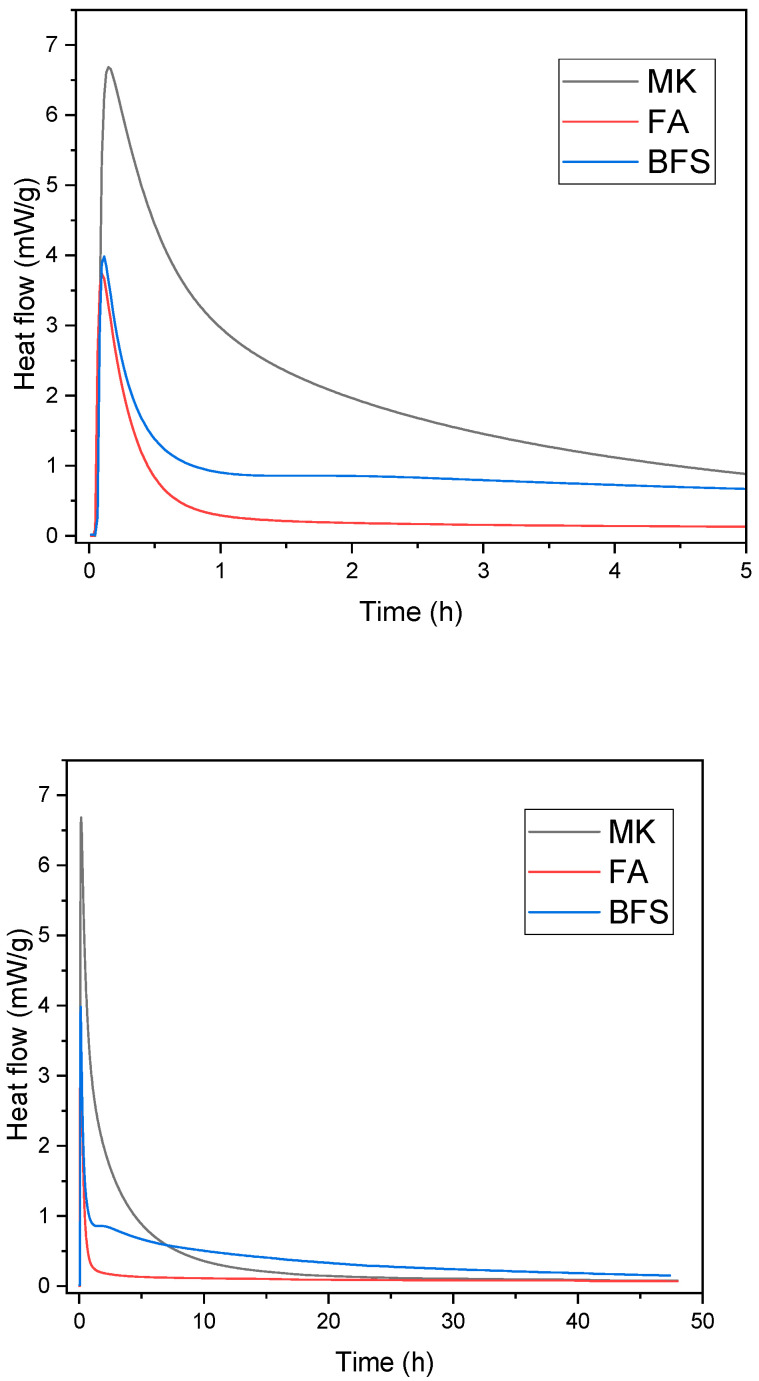
Calorimetry of geopolymers.

**Figure 10:**
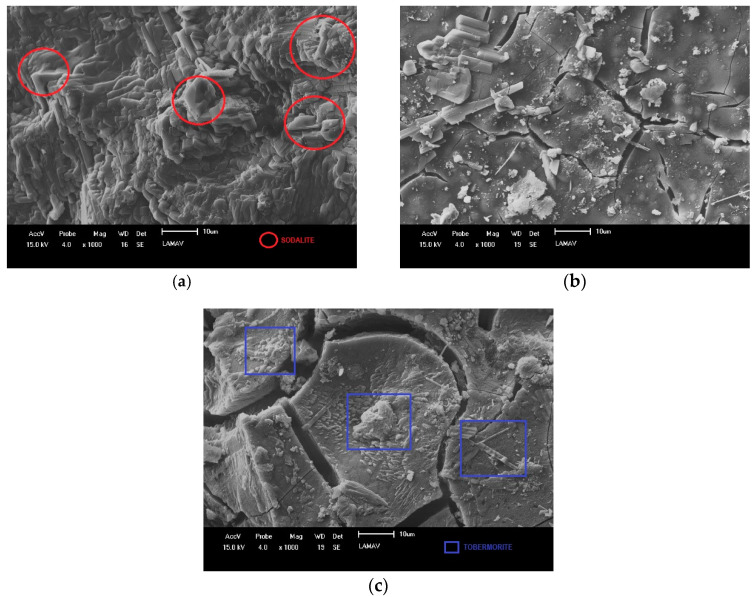
Scanning electron microscopy (SEM) of geopolymers: (**a**) MK—4.5; (**b**) FA—4.5; (**c**) BFS—4.5.

After analyzing the results in the fresh and hardened state, compositions MK—4.5, FA—4.5 and BFS—4.5 were chosen for a complementary analysis, through X-ray diffraction (Figure 8), calorimetry (Figure 9), and microscopy scanning electronics (Figure 10). Note that the MK—4.5 composition has sodalite and faujasite peaks [53,54]. These are the two most common types of zeolites in the alkaline activation process of binders rich in aluminosilicates, that is, in the geopolymerization process (Equation (1)). This information indicates that the MK—4.5 composition adequately completed the alkaline activation process, which explains the results shown in Figure 7 where compressive strength of approximately 30 MPa was obtained in thermal curing. In other words, the resistance results obtained by this composition are attributed to the presence of sodalite and faujasite. In the case of the FA—4.5 composition, a high amorphous band is observed, indicating that the alkaline activation reaction was very low, or almost non-existent for this material. This information explains the low mechanical strength results for the compositions containing fly ash. The strength values obtained are due only to physical principles of porosity reduction. In the case of the BFS—4.5 composition, sodalite and faujasite peaks are observed, but at a lower intensity than those observed in the MK—4.5 composition. In addition, the occurrence of tobermorite peaks is observed, a mineral typical of the alkaline activation of binders rich in calcium (Equation (2)). This indicates that the BFS—4.5 composition has the potential to further increase its compressive strength, since the formation of tobermorite is much slower than that of zeolites [55,56]. The compressive strength values of around 25 MPa obtained by thermally cured blast furnace slag (composition BFS—4.5) can be attributed to the presence of tobermorite. Therefore, the kinetics of the BFS—4.5 composition, although increased by thermal curing, is still lower than that of the MK—4.5 composition.

Therefore, based on the results in Figure 8, it is possible to observe that the binders with the best compressive strength results (Figure 7) are those that formed the largest quantity of crystals: tobermorite, in the case of blast furnace slag; and sodalite and faujasite, in the case of metakaolin. This suggests that crystallization or reduction of amorphism is a factor that is related to alkaline activation [36,57]. However, other factors, such as packing, can alter the compressive strength of alkali activated materials and must be analyzed appropriately.

It is important to describe the chemical mechanisms of the alkaline activation process: Equation (1) presents the formation of sodalite Na_6_(AlSiO_4_)_6_·4H_2_O, typically formed in the alkaline activation process of precursors rich in aluminosilicates, as is the case with metakaolin. We can see from the data in Table 2 that the binder contributes the reagents SiO_2_ (61.85%) and Al_2_O_3_ (32.81%). The activating solution contributes H_2_O and Na_2_O.

Equation (2) presents the formation of tobermorite Ca_5_Si_6_O_16_(OH)_2_[Na_2_O]·4H_2_O, where blast furnace slag contributes CaO (47.49%) and SiO_2_ (33.00%), as shown in Table 2. The activating solution contributes H_2_O and [Na_2_O] which is not part of the material’s crystallographic lattice, but rather occupies interstitial spaces balancing electrical charges, hence the symbols with brackets. Therefore, the results obtained in Figure 8 are consistent with the information in Table 2 and with the principles of alkaline activation.

(1) $$6SiO_{2}+3{Al}_{2}O_{3}+3{Na}_{2}O+4H_{2}O\to {Na}_{6}{\left(AlSiO_{4}\right)}_{6}\cdot 4H_{2}O$$

(2) $$5CaO+6SiO_{2}+[{Na}_{2}O]+5H_{2}O\to {Ca}_{5}{Si}_{6}O_{16}{\left(OH\right)}_{2}[{Na}_{2}O]\cdot 4H_{2}O$$

The calorimetry results (Figure 9) make it clear that the heat flux of the MK—4.5 composition is superior to the compositions with fly ash and blast furnace slag. The initial flux indicates approximately 6.5 mW/g for the MK—4.5 composition, while it presents a value of approximately 4.0 mW/g and 3.6 mW/g for the BFS—4.5 and FA—4.5 compositions, respectively. This first peak indicates that the metakaolin reaction is faster than that of the other binders. The results are consistent with those presented in Figure 8. This high flux in the first hours is related to the formation of zeolites, such as sodalite and faujasite, present in the MK—4.5 composition, which react more quickly than in the other binders. After the first peak, it is observed that the FA—4.5 composition presents a drop in the heat flux below 0.2 mW/g. This flux remains practically unchanged during the evaluation time shown in Figure 9, completed in 48 h, and is related to the non-alkaline activation of the material. In the case of the BFS—4.5 composition, it is observed that although the first heat peak is like that of the FA—4.5 composition and lower than the MK—4.5 composition, the observed flux drop is smaller than in the fly ash composition, being around 1 mW/g. However, between 5 and 10 h of testing, it is observed that the heat flux of the composition containing blast furnace slag exceeds the flow of the composition containing metakaolin. This is related to the tobermorite formation reaction, which is slower than the sodalite and faujasite formation reaction. At the end of the test, after 48 h, it is observed that the heat flux of the MK—4.5 and FA—4.5 compositions is practically the same, around 0.2 mW/g. However, the BFS—4.5 composition maintains a higher heat flux, indicating that the material is still reactive, albeit at a lower rate. These results confirm the information shown in Figure 8, that the blast furnace slag has the potential to present greater resistance at later ages, while the metakaolin has already reached a considerable level of reaction completion after 7 days of activation. On the other hand, fly ash does not present considerable heat flux, which may indicate that the material does not present a chemical principle as intense as that of the other binders. The strength acquired by the composition containing fly ash is mostly related to the physical principle of packing, which is why this precursor is most often used in conjunction with other binders, both as a pozzolanic material and in activated alkali materials.

Figure 10 shows the results of scanning electron microscopy (SEM) for the composition with 4.5 mol/L. Figure 10a shows the presence of a well-crystallized, compact phase with an irregular hexagonal shape. In other research, it has been observed that this morphology is typical of sodalite minerals, indicating a high level of alkaline activation, since the morphology of the material is very clear [6]. In the case of Figure 10b it is not possible to observe the presence of a clear crystalline phase, unlike Figure 10a. In Figure 10c it is possible to identify the presence of a phase in the form of an elongated rod. This structure refers to tobermorite, which agrees with previous results [31].

## 4. Conclusions

This article proposes the evaluation of the alkaline activation process of three classic binders (metakaolin, fly ash and blast furnace slag), in isolation. From the results, the conclusions of the research are:

- The characterization of the binders demonstrates that the blast furnace slag is rich in calcium, with alkaline activation similar to the hydration of Portland cement. Fly ash and metakaolin are rich in aluminosilicates, forming a geopolymerization process. Regarding granulometry, fly ash has smaller grains and metakaolin has more continuous granulometry, which may favor the activation process.
- Compositions containing metakaolin have higher viscosity, around 20–24 mPa·s, and lower workability. This is harmful to the behavior of the material, especially in solutions with higher molarity (5.00 and 5.50 mol/L) and may impair the properties of the hardened state. The compositions containing fly ash and blast furnace slag, in general, have equivalent consistencies, being more workable. Regarding the viscosity, the fly ash compositions do not present a defined pattern, which is related to the size of the grains of the material, smaller than the others, affecting the compactness. On the other hand, metakaolin and blast furnace slag show a clear tendency to increased viscosity with increasing molarity of the activator solution, a pattern found by other authors in the field.
- The results of density, water absorption and compressive strength tests, in normal and thermal curing, for metakaolin indicate that the results are optimized when using normal curing and molarity of 4.00 and 4.50 mol/L. This is evidenced by the occurrence of sodalite and faujasite in the alkaline activation process of the material. On the other hand, the greater reactivity of metakaolin induces porosity in thermal curing, reducing resistance and increasing water absorption. The use of solutions with molarity of 5.00 and 5.50 mol/L impairs the viscosity of the material, impairing compaction in the hardened state.
- The results of density, water absorption and compressive strength tests, in normal and thermal curing, for fly ash indicate that this precursor is efficient in material compactness, which is why it reduces water absorption. However, the resistance mechanisms of the material are not directly related to alkaline activation since the diffraction techniques did not detect the presence of crystalline peaks in the FA—4.5 composition. Thermal curing did not help the compressive strength of the material since no increase in this property was observed in this type of curing.
- The results of density, water absorption and compressive strength tests, in normal and thermal curing, for blast furnace slag indicate that the material presents more intense reactivity in thermal curing. This happens because the reaction kinetics of the material is low, but can be increased in alkaline media and with the effect of temperature. This is evidenced by the formation of sodalite and faujasite, at a lower intensity than the composition with metakaolin, but also due to the occurrence of tobermorite, whose reaction speed is lower than that of zeolites.

It can be concluded that the main objective of the research was achieved, which was to carry out a comparison between the alkaline activation process between different binders. Metakaolin proved to be reactive at room temperature; the alkaline activation process of blast furnace slag has intensified kinetics in thermal curing; and fly ash does not have alkaline activation potential when used as the only binder.

Finally, it is important to highlight that the results obtained in the research demonstrate the viability of producing activated alkali materials using metakaolin (normal curing) and blast furnace slag (thermal curing) as binders, with compressive strength results compatible with the usual applications of cementitious materials (mortar and concrete). This results in an alternative for the Portland cement industry, reducing environmental damage caused by CO_2_ emissions from the cement industry.

## Figures and Tables

**Table 1:** Studied compositions. Legend: MK—metakaolin; FA—fly ash; BFS—blast furnace slag.

Composition	Molarity (mol/L)	Metakaolin (g)	Fly Ash (g)	Blast Furnace Slag (g)	Sodium Silicate (g)	Sodium Hydroxide (g)	Water (g)
MK—4.0	4.0	145.80	0.00	0.00	35.00	16.30	71.00
MK—4.5	4.5	139.00	0.00	0.00	55.10	10.70	68.10
MK—5.0	5.0	131.95	0.00	0.00	72.60	6.10	64.40
MK—5.5	5.5	117.00	0.00	0.00	82.35	5.40	57.30
FA—4.0	4.0	0.00	145.80	0.00	35.00	16.30	71.00
FA—4.5	4.5	0.00	139.00	0.00	55.10	10.70	68.10
FA—5.0	5.0	0.00	131.95	0.00	72.60	6.10	64.40
FA—5.5	5.5	0.00	117.00	0.00	82.35	5.40	57.30
BFS—4.0	4.0	0.00	0.00	145.80	35.00	16.30	71.00
BFS—4.5	4.5	0.00	0.00	139.00	55.10	10.70	68.10
BFS—5.0	5.0	0.00	0.00	131.95	72.60	6.10	64.40
BFS—5.5	5.5	0.00	0.00	117.00	82.35	5.40	57.30

**Table 2:** Chemical composition of binders.

Precursor	SiO_2_	Al_2_O_3_	CaO	Fe_2_O_3_	TiO_2_	SO_3_	K_2_O	Others
Fly ash	69.20	22.00	0.65	6.5	-	0.50	0.95	0.20
Metakaolin	61.85	32.81	0.10	1.52	1.77	1.46	0.39	0.10
Blast furnace slag	33.00	9.69	47.49	0.58	0.86	0.95	0.45	6.98

**Table 3:** ANOVA (*p* ≤ 0.05) Randomized block design (Normal cure).

Source of Variation	Degrees of Liberty	Sum of Squares	Medium Squares	F Test	F Standard
Treatment	3	185.26	61.75	4.45	2.92
Blocks	2	239.79	119.89	8.65	3.32
Residue	30	415.90	13.86	-	-
Total	35	840.95	-	-	-

**Table 4:** ANOVA (*p* ≤ 0.05) Randomized block design (Thermal cure).

Source of Variation	Degrees of Liberty	Sum of Squares	Medium Squares	F Test	F Standard
Treatment	3	108.77	36.25	3.53	2.92
Blocks	2	152.50	76.25	7.42	3.32
Residue	30	308.41	10.28	-	-
Total	35	569.68	-	-	-

## Data Availability

Data are contained within the article.

## References

[1] Provis J.L. (2014). Geopolymers and other alkali activated materials: Why, how, and what?. Mater. Struct..

[2] Marvila M.T., de Azevedo A.R.G., Ferreira R.L.S., Vieira C.M.F., de Brito J., Adesina A. (2022). Validation of alternative methodologies by using capillarity in the determination of porosity parameters of cement-lime mortars. Mater. Struct..

[3] Papa E., Medri V., Paillard C., Contri B., Murri A.N., Vaccari A., Landi E. (2019). Geopolymer-hydrotalcite composites for CO_2_ capture. J. Clean. Prod..

[4] Komnitsas K., Zaharaki D. (2007). Geopolymerisation: A review and prospects for the minerals industry. Miner. Eng..

[5] Alonso M., Gismera S., Blanco M., Lanzón M., Puertas F. (2017). Alkali-activated mortars: Workability and rheological behaviour. Constr. Build. Mater..

[6] Marvila M.T., Azevedo A.R.G., Delaqua G.C.G., Mendes B.C., Pedroti L.G., Vieira C.M.F. (2021). Performance of geopolymer tiles in high temperature and saturation conditions. Constr. Build. Mater..

[7] Kul A., Ozel B.F., Ozcelikci E., Gunal M.F., Ulugol H., Yildirim G., Sahmaran M. (2023). Characterization and life cycle assessment of geopolymer mortars with masonry units and recycled concrete aggregates assorted from construction and demolition waste. J. Build. Eng..

[8] Munir Q., Abdulkareem M., Horttanainen M., Kärki T. (2023). A comparative cradle-to-gate life cycle assessment of geopolymer concrete produced from industrial side streams in comparison with traditional concrete. Sci. Total. Environ..

[9] Khater H.M., Ghareib M. (2021). Utilization of alkaline Aluminosilicate activation in heavy metals immobilization and producing dense hybrid composites. Arab. J. Sci. Eng..

[10] Nasir M., Johari M.A.M., Yusuf M.O., Maslehuddin M., Al-Harthi M.A., Dafalla H. (2019). Impact of Slag Content and Curing Methods on the Strength of Alkaline-Activated Silico-Manganese Fume/Blast Furnace Slag Mortars. Arab. J. Sci. Eng..

[11] Apolonio P.H., Lima J.S., Marinho E.P., Nobrega A.C.V., Freitas J.C.O., Martinelli A.E. (2020). Produção de geopolímeros utilizando cinza da casca de arroz como fonte complementar de sílica. Cerâmica.

[12] Amran Y.M., Alyousef R., Alabduljabbar H., El-Zeadani M. (2020). Clean production and properties of geopolymer concrete; A review. J. Clean. Prod..

[13] Görhan G., Kürklü G. (2022). Investigation of the effect of metakaolin substitution on physicomechanical properties of fly ash-based geopolymer mortars. Mater. Today Proc..

[14] Peng X., Li H., Hu Y. (2023). Preparation of metakaolin-fly ash cenosphere based geopolymer matrices for passive fire protection. J. Mater. Res. Technol..

[15] Tian X., Liu K., Yang X., Jiang T., Chen B., Tian Z., Wu J., Xia L., Huang D., Peng H. (2023). Synthesis of metakaolin-based geopolymer foamed materials using municipal solid waste incineration fly ash as a foaming agent. Waste Manag..

[16] de Carvalho T.A., Gaspar F., Marques A.C., Mateus A. (2024). Optimization of formulation ratios of geopolymer mortar based on metakaolin and biomass fly ash. Constr. Build. Mater..

[17] Cui C., Dang Y., Luo C., Wang L., Peng H. (2024). Mechanical Properties and Reaction Kinetics of Alkali-Activated Metakaolin. Materials.

[18] Marvila M.T., de Azevedo A.R.G., de Matos P.R., Monteiro S.N., Vieira C.M.F. (2021). Materials for Production of High and Ultra-High Performance Concrete: Review and Perspective of Possible Novel Materials. Materials.

[19] Mahakhud R., Priyadarshini M., Giri J.P. (2023). Utilization of ground granulated blast-furnace slag powder in brick industry: A new generation building material. Mater. Today Proc..

[20] Feng L., Yi S., Zhao S., Zhong Q., Ren F., Liu C., Zhang Y., Wang W., Xie N., Li Z. (2024). Recycling of Aluminosilicate-Based Solid Wastes through Alkali-Activation: Preparation, Characterization, and Challenges. Buildings.

[21] Federal Savings Bank (Brazil) (2023). Composition of unit costs. Sistema Nacional de Pesquisa de Custos e Índices da Construção Civil.

[22] (2016). Argamassa Para Assentamento e Revestimento de Paredes e Tetos—Determinação do Índice de Consistência.

[23] (2019). Cimento Portland―Determinação da Resistência à Compressão de Corpos de Prova Cilíndricos (In Portuguese).

[24] (2011). Argamassa e Concreto Endurecidos—Determinação da Absorção de Água, Índice de Vazios e Massa Específica.

[25] (2013). Standard Test Method for Heat of Hydration of Hydraulic Cement.

[26] He J., Zheng W., Bai W., Hu T., He J., Song X. (2021). Effect of reactive MgO on hydration and properties of alkali-activated slag pastes with different activators. Constr. Build. Mater..

[27] Gijbels K., Pontikes Y., Samyn P., Schreurs S., Schroeyers W. (2020). Effect of NaOH content on hydration, mineralogy, porosity and strength in alkali/sulfate-activated binders from ground granulated blast furnace slag and phosphogypsum. Cem. Concr. Res..

[28] Marvila M.T., de Azevedo A.R.G., de Oliveira L.B., Xavier G.d.C., Vieira C.M.F. (2021). Mechanical, physical and durability properties of activated alkali cement based on blast furnace slag as a function of %Na2O. Case Stud. Constr. Mater..

[29] Duxson P., Provis J.L. (2008). Designing precursors for geopolymer cements. J. Am. Ceram. Soc..

[30] Provis J.L., Van Deventer J.S. (2007). Geopolymerisation kinetics. In situ energy-dispersive X-ray diffractometry. Chem. Eng. Sci..

[31] Marvila M.T., de Azevedo A.R.G., de Matos P.R., Monteiro S.N., Vieira C.M.F. (2021). Rheological and the Fresh State Properties of Alkali-Activated Mortars by Blast Furnace Slag. Materials.

[32] Gencel O., Sutcu M., Erdogmus E., Koc V., Cay V.V., Gok M.S. (2013). Properties of bricks with waste ferrochromium slag and zeolite. J. Clean. Prod..

[33] Karakoç M.B., Türkmen I., Maraş M.M., Kantarci F., Demirboğa R. (2016). Sulfate resistance of ferrochrome slag based geopolymer concrete. Ceram. Int..

[34] Loganina V., Zhegera K., Fediuk R., Timokhin R., Zayakhanov M., Liseitsev Y. (2020). Amorphous Aluminosilicates as a Structure-Forming Additive in Cementitious Systems. J. Mater. Civ. Eng..

[35] Keppert M., Vejmelková E., Bezdička P., Doleželová M., Čáchová M., Scheinherrová L., Pokorný J., Vyšvařil M., Rovnaníková P., Černý R. (2018). Red-clay ceramic powders as geopolymer precursors: Consideration of amorphous portion and CaO content. Appl. Clay Sci..

[36] Vasić M.V., Terzić A., Radovanović, Radojević Z., Warr L.N. (2022). Alkali-activated geopolymerization of a low illitic raw clay and waste brick mixture. An alternative to traditional ceramics. Appl. Clay Sci..

[37] Marvila M.T., Azevedo A.R.G., Monteiro S.N. (2020). Verification of the application potential of the mathematical models of lyse, abrams and molinari in mortars based on cement and lime. J. Mater. Res. Technol..

[38] Shilar F.A., Ganachari S.V., Patil V.B., Khan T.Y., Khadar S.D.A. (2022). Molarity activity effect on mechanical and microstructure properties of geopolymer concrete: A review. Case Stud. Constr. Mater..

[39] Jithendra C., Dalawai V.N., Elavenil S. (2022). Effects of metakaolin and sodium silicate solution on workability and compressive strength of sustainable Geopolymer mortar. Mater. Today Proc..

[40] Ruviaro A.S., Santana H.A., Lima G.T.d.S., Barraza M.T., Silvestro L., Gleize P.J.P., Pelisser F. (2023). Valorization of oat husk ash in metakaolin-based geopolymer pastes. Constr. Build. Mater..

[41] Pundienė I., Pranckevičienė J., Zhu C., Kligys M. (2021). The role of temperature and activator solution molarity on the viscosity and hard structure formation of geopolymer pastes. Constr. Build. Mater..

[42] Koutník P., Soukup A., Bezucha P., Šafář J., Kohout J. (2020). Low viscosity metakaolinite based geopolymer binders. Constr. Build. Mater..

[43] Waqas R.M., Butt F., Zhu X., Jiang T., Tufail R.F. (2021). A Comprehensive Study on the Factors Affecting the Workability and Mechanical Properties of Ambient Cured Fly Ash and Slag Based Geopolymer Concrete. Appl. Sci..

[44] Sinkhonde D., Mashava D. (2022). Analysis of milling treatments of waste clay bricks effect on density and compressive strength of cement paste. Results Mater..

[45] de Azevedo A.R.G., Marvila M.T., de Oliveira L.B., Ferreira W.M., Colorado H., Teixeira S.R., Vieira C.M.F. (2021). Circular economy and durability in geopolymers ceramics pieces obtained from glass polishing waste. Int. J. Appl. Ceram. Technol..

[46] Liu X., Li S., Ding Y., Lu Z., Stephan D., Chen Y., Wang Z., Cui S. (2023). Investigation on admixtures applied to alkali-activated materials: A review. J. Build. Eng..

[47] Adesina A. (2022). Durability and microstructural characteristics of alkali activated materials made with waste glass as precursor: A review. Clean. Mater..

[48] Elzeadani M., Bompa D., Elghazouli A. (2022). One part alkali activated materials: A state-of-the-art review. J. Build. Eng..

[49] Li L., Xie J., Zhang B., Feng Y., Yang J. (2023). A state-of-the-art review on the setting behaviours of ground granulated blast furnace slag- and metakaolin-based alkali-activated materials. Constr. Build. Mater..

[50] Angulo-Ramírez D.E., de Gutiérrez R.M., Puertas F. (2017). Alkali-activated Portland blast-furnace slag cement: Mechanical properties and hydration. Constr. Build. Mater..

[51] Kretzer M.B., Effting C., Schwaab S., Schackow A. (2021). Hybrid geopolymer-cement coating mortar optimized based on metakaolin, fly ash, and granulated blast furnace slag. Clean. Eng. Technol..

[52] Li F., Liu L., Yang Z., Li S. (2021). Physical and mechanical properties and micro characteristics of fly ash-based geopolymer paste incorporated with waste Granulated Blast Furnace Slag (GBFS) and functionalized Multi-Walled Carbon Nanotubes (MWCNTs). J. Hazard. Mater..

[53] Wang Z., Huang Z., Zheng B., Wu D., Zheng S. (2022). Efficient removal of phosphate and ammonium from water by mesoporous tobermorite prepared from fly ash. J. Environ. Chem. Eng..

[54] Zhou Y., Zheng H., Li W., Ma T., Miao C. (2022). A deep learning potential applied in tobermorite phases and extended to calcium silicate hydrates. Cem. Concr. Res..

[55] Sudagar A.J., Andrejkovičová S., Rocha F., Patinha C., Velosa A., da Silva E.F. (2023). Compressive strength and heavy metal adsorption of cork residue, natural zeolite, and low-grade metakaolin-based geopolymers. Constr. Build. Mater..

[56] Yang S., Yang L., Gao M., Bai H., Nagasaka T. (2022). Synthesis of zeolite-geopolymer composites with high zeolite content for Pb(II) removal by a simple two-step method using fly ash and metakaolin. J. Clean. Prod..

[57] Boughriet A., Allahdin O., Poumaye N., Doyemet G., Tricot G., Revel B., Ouddane B., Wartel M. (2023). Alkali-Activated Brick Aggregates as Industrial Valorized Wastes: Synthesis and Properties. Ceramics.

